# Epidemiology, prenatal diagnosis, and neonatal outcomes of congenital heart defects in eastern China: a hospital-based multicenter study

**DOI:** 10.1186/s12887-020-02313-4

**Published:** 2020-09-02

**Authors:** Xiaohui Zhang, Yu Sun, Jiajun Zhu, Yuning Zhu, Liqian Qiu

**Affiliations:** 1 Department of Women’s Health, Women’s Hospital School of Medicine Zhejiang University, Hangzhou, 310006 Zhejiang Province China; 2 Department of Neonatology, Women’s Hospital School of Medicine Zhejiang University, Hangzhou, China; 3 Department of Serology Testing, Women’s Hospital School of Medicine Zhejiang University, Hangzhou, China

**Keywords:** Congenital heart disease, Epidemiology, Prenatal detection, Neonatal outcome

## Abstract

**Background:**

Congenital heart defect is the leading malformation in China. There may have been changes in congenital heart defect incidence because of birth policy shift in China over past years. This study aimed to investigate the epidemiology, prenatal diagnosis, and outcomes of congenital heart disease to improve medical and policy decisions.

**Methods:**

Data on cases of congenital heart disease identified during 2014–2018 were taken from the Zhejiang provincial birth defects surveillance system. Chi-square test, odds ratio (OR) and 95% confidence interval (CI) were used to explore epidemiology, prenatal diagnosis, and birth outcomes of congenital heart disease.

**Results:**

The average incidence of congenital heart disease was 16.0 per 1000 births, which increased by 62.2% during 2014–2018(χ^2^_trend_ = 181.41, *P* < 0.001). However, the average critical congenital heart incidence was 1.6 per 1000 births, which remained stable over time. Women aged ≤20 years (OR2.1, 95% CI 1.9–2.3) or ≥ 35 years (OR 1.2, 95% CI 1.2–1.3) were at higher risk of having babies with congenital heart disease than women aged 21–34 years. Women who gave birth in urban areas (OR 1.2, 95% CI 1.2–1.3), had a son (OR 1.3, 95% CI 1.3–1.4), or had multiple births (OR 4.0, 95% CI 3.7–4.4) were also at higher risk than those giving birth in rural areas, to girls, or single births, respectively. The three major subtypes of congenital heart disease were atrial septal defect (67.9%), patent ductus arteriosus (34.7%), and ventricular septal defect (6.4%). The prenatal detection rate of critical congenital heart disease was 90.0%, which was far higher than total congenital heart disease, at 22.2% (χ^2^ = 1687.67, *P* < 0.001). There were 1457 (17.1%) stillbirths, 106 (1.2%) early neonatal deaths, and 6983 (81.7%) live births associated with congenital heart disease.

**Conclusions:**

The high incidence of congenital heart disease in Zhejiang might be attributable to the large proportion of mild congenital heart disease. The incidence of critical congenital heart disease, the prenatal detection rate, and perinatal deaths from congenital heart disease are comparable to those in other studies.

## Background

Congenital heart disease (CHD) is frequently defined as structural or functional abnormalities of the heart or great vessels at birth. It has a number of subtypes, such as atrial septal defect (ASD), ventricular septal defect (VSD), patent ductus arteriosus (PDA), tetralogy of Fallot (TOF), and pulmonary stenosis et al. [1–5]. A meta-analysis of 1.3 million live births, with regional and specific CHD differences, showed that the global average prevalence of CHD at birth was 8.2 per 1000 births during 1970–2017 [4]. Critical CHD (CCHD) is any CHD requiring urgent medical intervention during early infancy, representing about 25% of babies with a CHD [1, 2, 6, 7].

CHD is the main cause of fetal and infant death. The 1-year infant survival rate from CHD has been found to range from around 75.0 to 97.0%, and is worse for CCHD [7–9]. Children living with CHD are associated with pneumonia, genetic or chromosomal abnormalities and the disease can have negative effects on their physical and mental health [5, 10, 11]. Infants with CCHD frequently require early surgical treatment after birth to improve outcomes [12, 13].

In China, CHD is one of the most common birth defects, and incidence varies from 7 to 22.9 per 1000 live births or perinatal infants [14–18]. China has a significant burden of CHD because of huge population. Over the past few years, the Chinese government has announced a birth policy shift [19, 20]. From November 2013, couples who either was an “only child” have been permitted to have a second child. Since the beginning of 2016, all couples have been allowed to have two children. Throughout the period of birth policy adjustment, there were some changes in characteristics of women having children and the associations of regarding CHD risk [21, 22]. It is therefore necessary to check the detailed epidemiology of CHD in China. Zhejiang Province is in eastern China, and the total population was approximately 57 million in 2018 [23]. This province is well-developed economically and maternal health is among the best in China [24]. Provincial hospital-based birth defect surveillance has been conducted in the province for more than 30 years. In this study, we comprehensively investigated the incidence, prenatal diagnosis, types and perinatal outcomes of CHD from 2014 to 2018 in Zhejiang Province, covering the period of changes in the birth control policy. Our findings may help to prevent CHD and promote infant health following the end of the one-child policy in China.

## Methods

### Birth defect surveillance system and study population

The hospital-based birth defects surveillance system in Zhejiang Province covers 30 regions and includes 90 hospitals, representing one third of the total births in this province each year. The registry system captures congenital anomalies in all births, including early fetal loss (death < 28 gestational weeks), stillbirth (fetal death at ≥28 gestational weeks), live birth, and neonatal death (death≤7 days after birth). Both singleton and multiple births are recorded.

A questionnaire is used for data collection by medical staff in surveillance hospitals. Information on the characteristics of women and their births, birth defect diagnosis, and perinatal outcomes are obtained from clinical records, laboratory records, mother and child healthcare books, and other sources. This surveillance system is web based. All cases with birth defect are reported to Women’s hospital, School of medicine Zhejiang University (provincial hospital). To ensure accuracy and completeness of identification of malformations, quality control is routinely performed according to the national birth defects surveillance program from community hospitals to provincial hospitals. We performed a cross sectional study, involving CHD reported in this surveillance system during 2014–2018.

### Antenatal care and CHD diagnosis

In Zhejiang, pregnant women normally receive their first antenatal health care check before 13 gestational weeks. They then receive five to seven more antenatal healthcare contacts, including prenatal screening and diagnosis. More antenatal healthcare visits are recommended for women at higher risk, such as those aged ≥35 years, with previous adverse pregnancy outcomes, or with abnormal findings in the current pregnancy. If the fetal nuchal translucency thickness is over 2.5 mm at a scan at 11 to 13 weeks, or there is a high risk of chromosomal abnormality based on serological screening, pregnant women are recommended to have echocardiography at 16 to 18 gestational weeks to detect possible CHD. In additional, all pregnant women are routinely provided with echocardiography between 24 and 28 weeks’ gestation, regardless of the findings during their first trimester screening. Prenatal findings are confirmed by pediatrician performing the postnatal diagnostics. For terminated fetus, autopsy is suggested, but not frequently used.

Newborn CHD screening has developed and strengthened remarkably over past years. In the study, neonates with positive prenatal echocardiography or abnormal heart auscultation were recommended with echocardiography during 2014–2017. Since 2018, all neonates have received routine pulse oximetry monitoring (by trained nurse) combined with heart auscultation (by pediatrician) in the first 24 to 48 h after birth. Those with positive records of prenatal echocardiography or with abnormal findings in CHD screening were offered neonatal ultrasound screening for CHD. The final diagnosis was based on neonatal echocardiographic findings from a scan and confirmed by pediatrician. Some patients with CHD had the diagnosis confirmed by a cardiologist through surgery or autopsy.

In this study, diagnoses of CHD were coded using the International Classification of Diseases version 10 (ICD-10) and subtypes were classified by codes Q20 to Q26. We excluded isolated patent foramen ovale and isolated PDA in preterm birth. We included ASD ≥3 mm and PDA ≥3 mm. We defined 12 types of CHD as CCHD, including: persistent truncus arteriosus (PTA, Q20.0), double-outlet right ventricle (DORV, Q20.1), d-transposition of the great vessels (DTGA, Q20.3), single ventricle (SV, Q20.4), TOF (Q21.3), pulmonary valve atresia (Q22.0), hypoplastic right heart (HRH, Q22.6), aortic valve stenosis (AoS, Q23.0), hypoplastic left heart syndrome (HLHS, Q23.4), coarctation of the aorta (COA, Q25.1), interrupted aortic arch (IAA, Q25.4), and total anomalous pulmonary venous return (TAPVR, Q26.2) using the definition from the International Clearing House for Birth Defects Surveillance and Research [12].

### Analysis

Data were electronically registered. SPSS 25.0 (IBM Corp, Armonk, New York, USA) was used for data analysis. The incidence of CHD was shown as the number of cases per 1000 births. Any type of CHD was calculated as the total incidence of CHD per 1000 births, with 95% confidence interval (CI) computed. We defined associated anomaly as CHD with non-cardiovascular malformation. Continuous variables were shown as mean and standard deviation (SD), and categorical variables as number and percentage. Chi-square trend analysis was used to track changes in the incidence of CHD over time. Crude odds ratio (OR) was calculated to examine the risk factors for CHD. *P* values < 0.05 (two-sided) were considered statistically significant, unless indicated otherwise.

## Results

### Patients’ characteristics and risk factors for CHD

During the study period, 8546 of 534,002 births were identified as having CHD, giving an average incidence of CHD of 16.0 per 1000 births (95% CI 15.69–16.32). The mean age of the mothers was 28.9 ± 5.1 years (range: 15–50 years). Younger (OR 2.11, 95% CI 1.88–2.31) or older women (OR 1.25, 95% CI 1.18–1.33) were at higher risk of having a child with CHD than those aged 21–34 years (both *P* < 0.001). Births in urban areas (OR 1.24, 95% CI 1.18–1.31), male babies (OR1.34, 95% CI 1.28–1.40), and multiple births (OR4.03, 95% CI 3.70–4.40) were also associated with increased risk of CHD compared with births in rural areas, female babies, and single births, respectively (all *P* < 0.001, Table 1).

**Table 1:** Patients’ characteristics and risk factors for CHD

Variable	Births (n)	CHD (n)	Incidence (per 1000 births)	OR value and 95% CI	*P* value
Maternal age (years)					
< 20	9879	312	31.8	2.11(1.88–2.31)	< 0.001
21–34	456,027	6944	15.3	Ref	
≥35	68,096	1290	18.9	1.25(1.18–1.33)	< 0.001
Area					
Urban	320,029	6343	19.8	1.24(1.18–1.31)	< 0.001
Rural	138,754	2203	15.9	Ref	
Birth gender					
Male	253,015	4614	18.2	1.34(1.28–1.40)	< 0.001
Female	280,921	3843	13.7	Ref	
Unknown	–	65	–	–	–
Singleton	524,108	7965	15.2	Ref	
Multiple birth	9894	581	58.7	4.03(3.70–4.40)	< 0.001

### Time trends and subgroups of CHD

The overall incidence of CHD increased from 12.7 per 1000 births in 2014 to 20.6 per 1000 births in 2018, an increase of 62.2% (χ^2^_*trend*_ = 181.41, *P* < 0.001). In total, 9.9% (842) cases were CCHD, with an average incidence of 1.6 per 1000 births (95% CI 1.47–1.69). However, there was no significant time trend in the incidence of CCHD (χ^2^_*trend*_ = 0.26, *P* = 0.609, Table 2).

**Table 2:** Trends in the incidence of CHD and CCHD during 2014–2018 (per 1000 births)

Time	Birth Number	Critical CHD			Total CHD		
		Number of CCHD	No-CCHD	CCHD(‰)	Number of CHD	No-CHD	CHD (‰)
2014	107,639	164	107,475	1.5	1363	106,276	12.7
2015	91,423	171	91,252	1.9	1496	89,927	16.4
2016	119,976	188	119,788	1.6	1670	118,306	13.9
2017	114,545	146	114,399	1.3	1951	112,594	17.0
2018	100,419	173	100,246	1.7	2066	98,353	20.6
total	534,002	842	533,160	1.6	8546	525,456	16.0
χ^2^_*trend*_		0.26			181.41		
*P*		0.609			< 0.001		

**Fig. 1:**
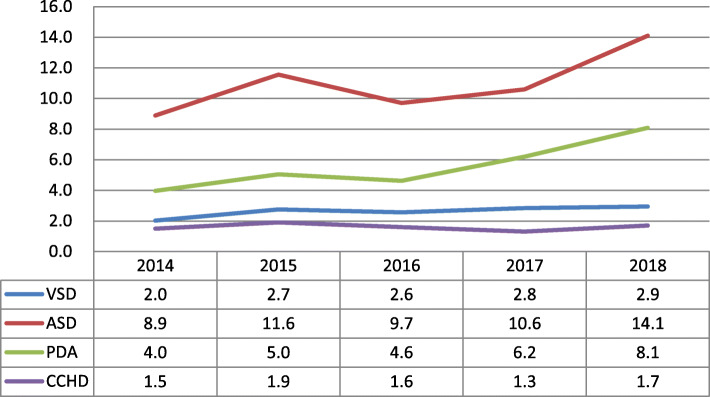
the incidences of CCHD, VSD, ASD and PDA over time (per 1000 births). *CCHD, Critical CHD; VSD, Ventricular septal defect; ASD, Atrial septal defect; PDA, Patent ductus arteriosus

There were associated anomalies in 13.7% (1167/8546) of all CHD cases and 24.3% (245/1007) of CCHD cases (χ^2^ = 81.49, *P* < 0.001). The three most frequent subtypes of CHD were ASD (67.9%), PDA (34.7%), and VSD (16.4%) (Table 3). From 2014 to 2018, the incidence of ASD (χ^2^_*trend*_ = 86.47, *P* < 0.001), PDA (χ^2^_*trend*_ = 165.23, *P* < 0.001) and VSD (χ^2^_*trend*_ = 13.00, *P* < 0.001) increased by 58.4, 102.5 and 45.0%, respectively (Fig. 1).

**Table 3:** Rank of subtypes of CHD by incidence

Rank	Subgroup	N	Incidence Per 1000 birth	Proportion (%)
1	ASD/Q21.1	5807	10.9	67.9
2	PDA/Q25.0	2963	5.5	34.7
3	VSD/Q21.0	1398	2.6	16.4
4	AVSD/Q21.2	368	0.7	4.3
5	TOF/Q21.3	269	0.5	3.1
6	IAA/Q25.4	131	0.2	1.5
7	Pulmonary stenosis/Q25.6	113	0.2	1.3
8	DORV/Q20.1	108	0.2	1.3
9	SV/ Q20.4	88	0.2	1.0
10	DTGA/Q20.3	90	0.2	1.1
11	HLHS / Q23.4	65	0.1	0.8
12	COA/Q25.3	64	0.1	0.7
13	PTA/ Q20.0	68	0.1	0.8
14	Dextrocardia/Q24.0	61	0.1	0.7
15	HRH/ Q22.6	40	0.1	0.5
16	Pulmonaryvalve atresia/Q22.0	27	0.1	0.3
17	AoS/Q23.0	9	< 0.1	0.1
18	TAPVR/Q26.2	8	< 0.1	0.1

*ASD* Atrial septal defect, *PDA* Patent ductus arteriosus, *VSD* Ventricular septal defect, *AVSD* Atrioventricular septal defect, *TOF* Tetralogy of Fallot, *IAA* Interrupted aortic arch and others, *DORV* Double outlet right ventricle, *SV* Single ventricle, *DTGA* d-Transposition of great vessels, *HLHS* Hypoplastic left heart syndrome, *COA* Coarctation of the aorta, *PTA* Persistent truncus arteriosus, *HRH* Hypoplastic right heart, *AoS* Aortic valve stenosis, *TAPVR* Total anomalous pulmonary venous return

### Prenatal detection and outcomes of CHD

In total, 22.8% (1949/8546) of CHD cases were prenatally detected at a mean gestational age of 25.7 ± 5.2 weeks. The prenatal detection rate was 90.0% (758/842) for CCHD, which was far higher than that for overall CHD cases (χ ^2^ = 1687.67, *P* < 0.001). Table 4 shows the levels of prenatal detection of CHD. SV, hypoplastic left heart syndrome, and double-outlet right ventricle were the top three prenatal diagnosis subgroups of CHD. The proportions (number) of prenatally-diagnosed CHD cases identified in the first, second and third trimesters were 1.2% (23), 72.8% (1420) and 26.0% (506), respectively. Of cases diagnosed in the first trimester, 87.0% (20) had associated non-cardiovascular malformations and 26.1% (6) had at least two types of CHD. The figures for combined non-cardiovascular and cardiovascular malformations were 36.1% (512) and 46.2% (656), respectively, for CHD diagnosed in the second trimester, and 31.03% (157) and 43.7% (221), respectively, for those diagnosed in the third trimester.

**Table 4:** Rank of prenatal detection subgroups of CHD

Rank	Subgroup	Prenatal detection	Proportion (%)
1	SV/ Q20.4	87	98.9
2	HLHS/ Q23.4	64	98.5
3	DORV/Q20.1	105	97.2
4	PTA/ Q20.0	64	94.1
5	HRH/ Q22.6	37	92.5
6	TOF/Q21.3	245	91.1
7	COA/Q25.3	58	90.6
8	Pulmonary stenosis/Q25.6	101	89.4
9	AoS/Q23.0	8	88.9
10	Dextrocardia/Q24.0	53	86.9
11	Pulmonaryvalve atresia/Q22.0	23	85.2
12	IAA/Q25.4	112	85.5
13	DTGA/Q20.3	72	80.0
14	TAPVR/Q26.2	5	62.5
15	AVSD/Q21.2	205	55.7
16	VSD/Q21.0	649	46.4
17	PDA/Q25.0	121	4.1
18	ASD/Q21.1	206	3.5

*SV* Single ventricle, *HLHS* Hypoplastic left heart syndrome, *DORV* Double outlet right ventricle, *PTA* Persistent truncus arteriosus, *HRH* Hypoplastic right heart, *TOF* Tetralogy of Fallot, *COA* Coarctation of the aorta, *AoS* Aortic valve stenosis, *IAA* Interrupted aortic arch and others, *DTGA* d-Transposition of great vessels, *TAPVR* Total anomalous pulmonary venous return, *AVSD* Atrioventricular septal defect, *VSD* Ventricular septal defect, *PDA* Patent ductus arteriosus, *ASD*, Atrial septal defect

In total, 17.1% (1457) of stillbirths, 1.2% (106) of early neonatal deaths and 81.7% (6983) of live births were associated with CHD. Of prenatally diagnosed CHD, 74.7% were terminated (1456/1949).

## Discussion

The study describes the epidemiology of CHD in eastern China. This multicenter study with a large sample size could accurately reflect occurrence of CHD, including some rare subtypes. We observed an upward trend in incidence of total CHD over time, but the incidence of CCHD did not change significantly. ASD, PDA and VSD were the predominant subtypes of CHD. The prenatal detection rate of CHD differed by category. Over 95% of cases of SV, HLHS, and DORV were prenatally diagnosed. More than 80% of patients with CHD were born alive. The estimated risk of CHD increased in births to women who were younger (< 20 years) or older (≥ 35 years), in urban areas, with male babies, and in multiple births.

The increasing incidence of CHD in our study is consistent with most previous reports in China and other countries [4, 15, 21, 22]. The global incidence of CHD increased from 4.6 per 1000 live births in 1970–1974 to 9.4 per 1000 live births in 2010–2017 [4]. Increasing trends have also been observed for some specific forms of CHD, such as ASD in the USA and SV, ASD, and TOF in Europe [1, 25]. The increase in incidence of CHD in our study might reflect a true increase. However, better CHD screening, technology, and follow-up may also have contributed to the increase observed in both Guangdong Province in China and in Europe [15, 25]. Since birth policy adjustment, changes of maternal characteristics could also partly explain the upward trend of total CHD [22]. There was a strengthen in neonatal CHD screening strategy in 2018 in China, and the incidence of CHD increased then compared with 2017 and before. Fortunately, we did not find any significant time trend in the incidence of CCHD throughout the study period. The substantial increase in incidence of CHD may have been for a rise in detection of ASD, PDA and VSD. This confirmed previous findings at the global level that 93.4% of the increase in incidence of CHD was because of better detection of VSD, ASD, and PDA, which is also similar to results in middle-income countries, and Guangdong province of China [4, 15, 26].

The total incidence of CHD reached 20.57 per 1000 births in 2018, which is much higher than in most previous studies (8–10 per 1000 births at global and local levels) [4, 14–17]. However, the rate of CHD was similar to that in southern Israel (24.6 per 1000 live births) and Langfang in China (22.9 per 1000 live births) [18, 27]. Differences in study populations, prenatal detection capability, and ascertainment of criteria might explain the heterogeneity of CHD incidence. Population-based studies on CHD by EUROCAT and in the USA had a long-term follow-up to at least 1 year of life or without age limitations [2, 3]. In Shanxi, isolated patent foramen ovale and PDA < 28 days of life were not analyzed [14]. In Guangdong, ASD was determined as < 5 mm; the final diagnosis for fossa ovalis, PDA, or patent foramen ovale was at 6 months after birth; and neonates ≤28 gestational weeks were excluded [15]. In Southern Israel, the high occurrence of CHD was possibly due to a strict protocol for early diagnosis of CHD and a high rate of consanguinity in local women [27]. Data from our study were hospital-based and patients were followed up within 7 days of birth. We included both ASD and PDA at and over 3 mm in diameter. Our study might therefore have overestimated CHD compared with previous studies. However, we found a similar or even slightly lower incidence of CCHD compared with the National Birth Defects Prevention Network and the International Clearing House for Birth Defects Surveillance and Research based on 12 countries (approximately 1.9 per 1000 births) [1, 12]. The main threat from CHD in our study was therefore not severe lesions.

In this study, prenatal detection rates varied with CHD categories. In earlier literatures, the prenatal detection rate ranged from under 10% to approximately 100% for specific CHD, whereas CCHD was more easily identified before delivery than noncritical defect [9, 12, 16, 28–32]. In the UK, more than 50% of CHD diagnoses are made or suspected during a scan in the first trimester [32]. However, only a few patients were diagnosed with CHD in the first trimester in our study. The prenatal detection rate of CHD depends on development of technology, as well as the distribution of subgroups of CHD. The large proportion of ASD, a relative low proportion of combined malformation in our study might have led to delay in prenatal detection. For example, 37.1% cases of CHD had chromosomal anomaly or non-cardiovascular malformation in a Danish study, and the prenatal detection rate was approximately 30% [30]. Nevertheless, some frequently-seen types of CCHD, such as HLHS, TOF, and DTGA, had a comparable or even higher detection rate than in most previous studies [30–32]. Of prenatally diagnosed CHD, the proportion for termination was 74.7%, which was similar to Hunan in China [16]. For different countries, differences in legal requirements for termination should be considered. In view of the outcomes for CHD, a long-term follow-up is required because most previous studies focused on at least 1-year mortality [6–8].

Advanced maternal age is a risk factor for CHD [16, 33]. However, data on the effect of younger maternal age on CHD are limited. One study in Shenzhen in China showed that maternal age < 25 years reduced the risk of CHD [34]. This issue should be investigated in greater detail. Older women or those pregnant during adolescence should be focused on and provided with advice about their health. A higher incidence of CHD was found in urban areas in our study, which is similar to a study in Langfang and Hunan [16, 18]. Women in urban areas may have easy access to high-quality antenatal health care, which can lead to a higher detection rate of CHD. However, environmental exposure and social pressure in urban life may also have a cooperative effect. The association between sex and CHD is not consistent across studies, and the reasons for this inconsistency remain elusive [14, 18]. Some studies indicated sex dominance differed by CHD category [9, 26].

This study had two main limitations. First, we did not consider the effects of the social or natural environment, and other maternal complications because of limited data [5, 14, 34, 35]. Second, we could not compare ASD for different criteria and we had no precise data about the diameter of ASD. Children with symptomatic ASD have higher morbidity than those with no symptoms, so future studies should provide further analysis by risk of ASD [36].

## Conclusions

A much higher incidence of CHD was observed in Zhejiang than widely reported. This was greatly contributed to a large proportion of mild CHD. The prenatal detection rates of CHD differed by categories. CCHD and CHD with associated malformations were more likely to be confirmed prenatally. Older or younger women, male births, births in urban should be noticed. The findings suggested target strategic plan for CHD prevention and intervention.

## Data Availability

Data were available on reasonable demand contacting with corresponding author.

## Abbreviations

CHD: Congenital heart disease
CCHD: Critical CHD
OR: Odds ratio
CI: Confidence interval
ASD: Atrial septal defect
PDA: Patent ductus arteriosus
VSD: Ventricular septal defect
PTA: Persistent truncus arteriosus
DORV: Double outlet right ventricle
SV: Single ventricle
DTGA: d-Transposition of great vessels
AVSD: Atrioventricular septal defect
TOF: Tetralogy of Fallot
HRH: Hypoplastic right heart
HLHS: Hypoplastic left heart syndrome
AoS: Aortic valve stenosis
COA: Coarctation of the aorta
IAA: Interrupted aortic arch and others
